# Development of Capillary Zone Electrophoresis Method for the Simultaneous Separation and Quantification of Metformin and Pioglitazone in Dosage Forms; and Comparison with HPLC Method

**DOI:** 10.3390/molecules28031184

**Published:** 2023-01-25

**Authors:** Maymonah K. I. AlThikrallah, Abubakr M. Idris, Abdalla Ahmed Elbashir, Rafea E. E. Elgorashe, Alyah Buzid, Ahmed O. Alnajjar

**Affiliations:** 1 Department of Chemistry, College of Science, King Faisal University, Al-Ahsa 31982, Saudi Arabia; 2 Department of Chemistry, College of Science, King Khalid University, Abha 62529, Saudi Arabia; 3 Research Center for Advanced Materials Science (RCAMS), King Khalid University, Abha 62529, Saudi Arabia

**Keywords:** capillary electrophoresis, pioglitazone, metformin, diabetes mellitus, pharmaceutical formulations, analytical method validation

## Abstract

A capillary zone electrophoretic (CZE) method was developed, validated, and applied for the assay of metformin (MET) and pioglitazone (PIO) in pharmaceutical formulations. The optimum running buffer composition was found to be 75 mmol/L phosphate buffer containing 30% acetonitrile (ACN) at pH 4.0. The optimum instrumental conditions were found to be injection time, 10 s; applied voltage, 25 kV; hydrodynamic injection pressure, 0.5 psi for 10 s, capillary temperature, 25 °C; and the detection wavelength, 210 nm. The quantifications were calculated based on the ratio of the peak areas of analytes to atenolol as an internal standard. The CZE method was validated in terms of accuracy (98.21–104.81%), intra- and inter-day precision of migration time and peak area (relative standard deviation ≤ 5%), linearity (correlation coefficients ≥ 0.9985), limit of detection (≤0.277 μg/mL), and limit of quantitation (≤0.315 μg/mL). The proposed method was applied for the analysis of PIO and MET both individually and in a combined dosage tablet formulation. All electrophoretic parameters were calculated and evaluated. A previously reported high-performance liquid chromatographic (HPLC) method was also applied to the same samples. A comprehensive comparison was then carried out for the analytical features of both methods CZE and HPLC. Comparable results were obtained with the advantage of reagent consumption and separation efficiency of CZE over HPLC and shorter analysis time by HPLC compared with CZE.

## 1. Introduction

Diabetes mellitus is a serious metabolic disorder characterized by high blood glucose levels and occurred when the body is not able to produce enough insulin or cannot use insulin effectively [1,2]. It has been demonstrated that hyperglycemia induces cellular oxidative stress in diabetes due to the imbalance of production between antioxidant and radical species [3]. Thus, diabetics are at high risk of developing life-threatening diseases such as cardiovascular disease and cancers, resulting in increased mortality [4]. Diabetes is developed due to the risk factor including overweight, sedentary lifestyle, and genetic susceptibility [5]. International Diabetes Federation (IDF) reported that approximately 537 million adults (20–79 years) worldwide suffer from diabetes in 2021 and is expected to reach 643 million by 2030 and 783 million by 2045. The World Health Organization (WHO) reported that Saudi Arabia is the second-largest diabetic population in the Middle East and the seventh-largest in the world [6]. The three main types of diabetes are type 1 diabetes (T1D), type 2 diabetes (T2D), and gestational diabetes mellites (GDM). T1D is due to the destruction of β-cell insulin secretion and leads to insulin deficiency, whereas T2D is due to the loss of β-cell insulin secretion. GDM occurs in the second or third trimester of pregnancy that was not diagnosed before gestation [7]. Therefore, medication instead of insulin must be used for the treatment of T2D.

**Figure 1:**
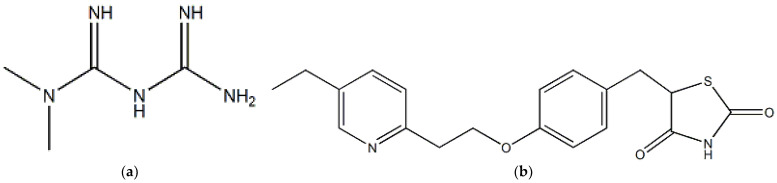
Chemical structures of (**a**) metformin and (**b**) pioglitazone.

Chemically, metformin (MET) is 1,1-dimethyl biguanide (Figure 1a). It is an agent used for the treatment of T2D with effective antihyperglycemic properties attributed to the suppression of hepatic glucose production and enhancing peripheral glucose uptake [8]. The American Diabetes Association (ADA) and the European Association for the Study of Diabetes (EASD) recommended that MET is the first-line treatment option for T2D [9]. In vivo and vitro studies have indicated that MET has antioxidant properties including the suppression of the formation of advanced glycation end products (AGEs) that enhance the production of cellular oxidative stress [10,11] and lower the concentration of methylglyoxal, a precursor of AGEs [12].

It is recommended to use a second agent along with MET if patients suffer from a high level of glycated hemoglobin (HbA_1c_) of ≥9 or glycemic target is not achieved after three months of treatment with MET [13]. Pioglitazone (PIO) belongs to the thiazolidinedione group. It is chemically (RS)-5-(4-[2-(5-ethylpyridin-2-yl)ethoxy] benzyl)thiazolidine-2,4-dione (Figure 1b). PIO is beneficial in targeting insulin resistance, reducing cardiovascular risk, and improving the overall lipid profile [13].

The literature reveals that various separation methods were used for the individual determination of MET [14] and with other diabetic drugs [15,16,17]. In addition, several chromatographic methods were reported for the determination of PIO, either alone [18,19] or with its main metabolites [20,21], besides in combination with other drugs [22,23,24].

In another context, since its introduction of its modern version, capillary zone electrophoresis (CZE) has evolved into a highly mature analytical technique. After its instrument commercialization, CZE has taken its place among well-established analytical techniques, in general, and separation techniques, in particular [25,26,27,28]. This is because of its benefits in terms of versatility, fastness, very low consumption of reagents, and organic solvents. The research field of the applications of CZE remains active, which focus on presenting alternative methods to chromatographic techniques [25,26,27]. The application of CZE in pharmaceutical analysis was reported in many fields such as chiral separation [29], analysis of pharmaceutical active ingredient in single and in combination formulation [25,26,27], and in determination of pharmaceutical impurity [30].

In this context, CZE was exploited for the assay of MET [26,31,32,33] and PIO [34] and with other drugs or metabolites. Nevertheless, to our knowledge, no CZE method for the assay of PIO and MET in combination dosage form has been reported. Based on the aforementioned discussion, it has been proposed to benefit the advantages of CZE for developing a new method for quality control purpose. The proposed method was validated according to International Council on Harmonisation of Technical Requirements for Registration of Pharmaceuticals for Human Use (ICH) guidelines. The calibration ranges, accuracy, intra- and inter- day precision, limits of detection (LOD), and limits of quantification (LOQ) were all examined. The method was also applied to real pharmaceutical formulations. The analytical figure of merits of the CZE method were compared with a previously reported reversed phase high-performance liquid chromatography (RP-HPLC) method [35].

## 2. Results and Discussion

### 2.1. CZE method Development and Optimization

Primarily, spectrum scans of both drugs MET and PIO were carried out by a UV/Vis spectrophotometer. The maximum absorbance of MET and PIO were detected at 210 nm. Therefore, the PDA detector coupled with the CZE system was set at 210 nm for the quantification of both MET and PIO. In addition, the preliminary investigation also showed that phosphate buffer recorded better electropherograms, in terms of resolution, migration time, peak shape, peak height, baseline noise, and the electric current produced, than borate and acetate buffers. The literature also reported that the use of phosphate buffer was appropriate for MET [31,36] and PIO [34], with other analytes, assay methods.

**Figure 2:**
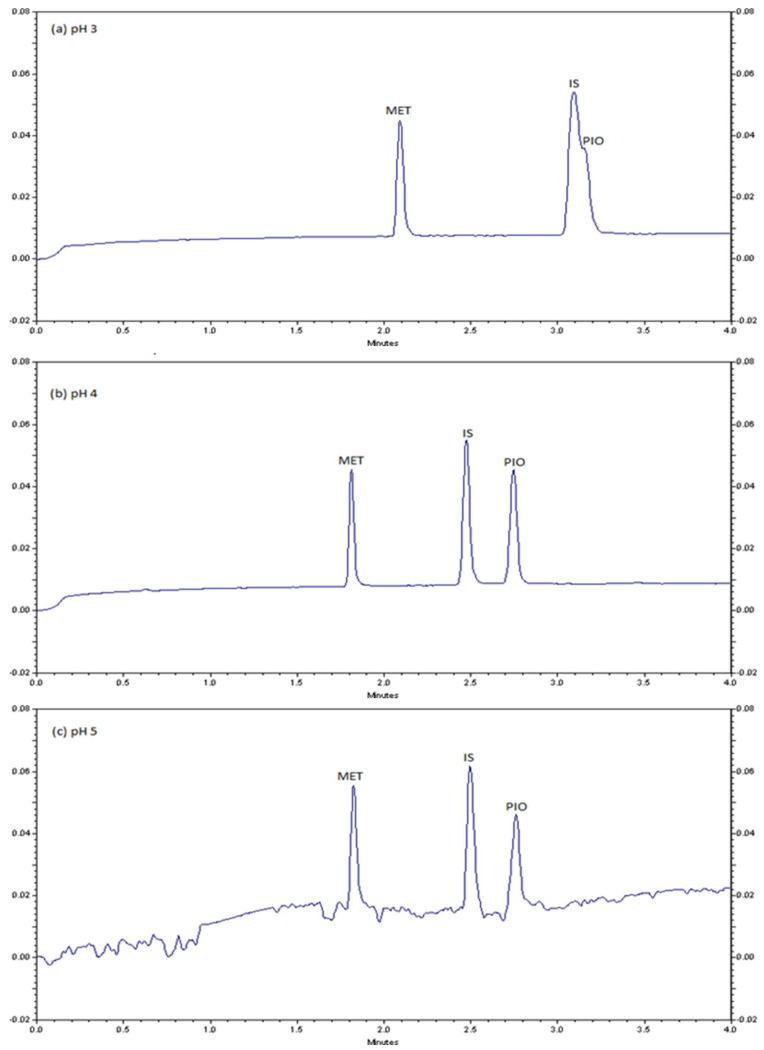
Typical electropherograms using different pH levels ((**a**) pH 3, (**b**) pH 4, and (**c**) pH 5 for the separation of 75 µg/mL metformin (MET), 75 µg/mL internal standard (IS), and 50 µg/mL pioglitazone (PIO). Separation conditions: capillary column dimension 37 cm × 52 μm, 75 mmol/L sodium phosphate buffer, 10 s hydrodynamic injection time at 0.5 psi pressure, 25 kV separation voltage, 25 °C column temperature, UV detection at 210 nm.

In separation of ionizable molecules by CZE, pH has a significant role, as it controls the degree of ionization of solutes [37]. The acidity of the buffer may affect mobility and electroosmosis flow (EOF) by altering the dissociation constant of analyte and Si-OH groups on the capillary [37]. Based on the pKa values of MET (12.4) [38] and PIO (5.6) [34], both compounds are positively charged under acidic conditions and can be analyzed by CZE. Notably, atenolol was chosen as an internal standard (IS) because it has a pKa value of 9.6. Its molecular weight is more than MET and less than PIO. Hence, it is positively charged, and eluted after MET and before PIO. Therefore, the effect of pH in the range of 3.0–5.0 was examined. The results demonstrate that the migration times were decreased with increasing pH as illustrated in Figure 2. pH 4 was determined as the optimum in terms of resolution and peak symmetry. At pH 4, the analytes are protonated and migrated toward the cathode electrode due to the electrophoretic mobility. Below pH 4, PIO peak overlapped with the IS, while above pH 4, the baseline is noisy, and the results are irreproducible. It is known that pH controls the degree of ionization of the analyte, its elelectrophoretic mobility, and the magnitude of the EOF. Particularly, at high pH, acidic silanol groups on the inside of the capillary as well as acidic group of atenolol are dissociated that may lead to high noise level [39].

The buffer concentration also plays an important role in controlling the EOF and the current produced in the capillary, baseline stability, and peak shape that affect affects separation efficiency. In the current study, the examined phosphate buffer concentrations were 20, 50, and 75 mmol/L. Better resolution and baseline was achieved with 75 mmol/L phosphate buffer concentration, therefore it was chosen as the optimum.

**Figure 3:**
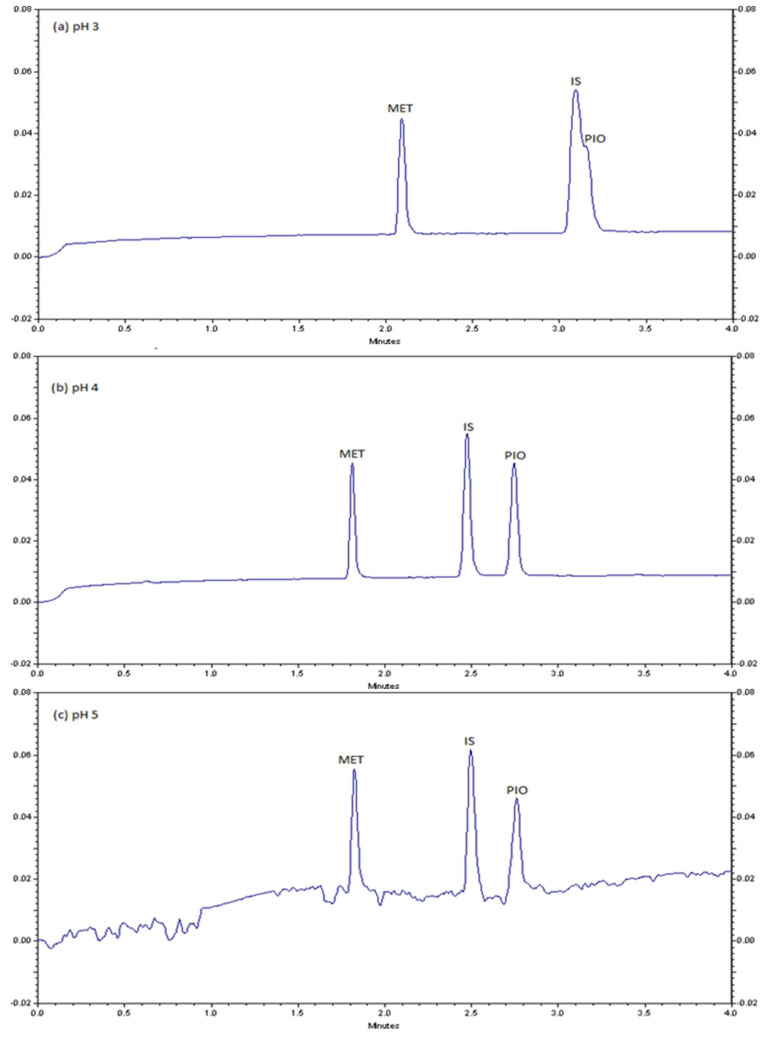
Typical electropherograms for application of the proposed capillary zone electrophoresis method (**a**) for the determination of 75 µg/mL metformin (MET) in pharmaceutical formulation (Glucophage^®^), (**b**) 30 µg/mL pioglitazone (PIO) in pharmaceutical formulation (Actos^®^) and (**c**) for 75 µg/mL metformin (MET), and 30 µg/mL pioglitazone (PIO) in synthetic mixture, in all cases 50 µg/mL internal standard (IS), was used.

Furthermore, the addition of an organic solvent to the running buffer was experimented because of its effect on viscosity, dielectric constant, and zeta potential, in addition to the low solubility of PIO in a buffered aqueous solution. Notably, ACN and methanol are recommended as non-aqueous solvents in CZE. Such solvents manipulate separation selectivity. The lower currents in non-aqueous solvents enable wide bore capillaries, which allows larger sample load, and enables the use of high electric field strengths. Detection sensitivity, in many cases, can also be enhanced [40]. Different ACN ratios (20, 30, and 40%) were examined at fixed conditions of 75 mmol/L phosphate buffer, pH 4.0, 10 s hydrodynamic injection time at 0.5 psi pressure, 25 kV separation voltage, 25 °C column temperature, and UV detection at 210 nm. The electropherograms are illustrated in Figure 3. It was observed that the addition of ACN increased solubility of PIO. However, 30% ACN ratio recorded the best peak shape, which was set as the optimum.

For other instrumental conditions, voltage is an important parameter in CZE that greatly affects in both migration times and resolution. The effect of voltage on electrophoretic parameters was studied at 15, 20, and 25 kV. It was noticed that high applied voltage increases the EOF and thus leads to shorter migration time and higher efficiency. As a result, a voltage of 25 kV was set as the optimum. In addition, temperature of the capillary column affects the viscosity of the buffer and thus affects EOF and electrophoretic mobilities. The effect of temperatures on separation was investigated at 20, 25, and 30 °C. The 25 °C was chosen as the optimum temperature in terms of resolution and analysis time. Moreover, the change in injection time leads to the change in the height and width of the peaks. In the current study, the samples were injected hydrodynamically at 0.5 psi in different injection times 5.0, 10.0, and 15.0 s. By comparing the obtained results, it was found that at 5.0 s the peaks intensity is very low while at 15.0 s the peak of PIO was expanded and deformed. 10.0 s showed the best result and therefore it was set as the optimum injection time.

From the above experiments, the optimum running buffer composition for the simultaneous analysis of MET and PIO was 75 mmol/L phosphate buffer containing 30% ACN at pH 4.0. On the other hand, the instrumental optimum conditions were injection time, 10 s; applied voltage, 25 kV; capillary temperature, 25 °C; and the detection wavelength, 210 nm.

### 2.2. CZE Method Validation

The proposed CZE method was validated with respect to linearity, limit of detection (LOD), limit of quantification (LOQ), precision, and accuracy. In this context, the calibration curves were plotted using calibrator concentrations against the peak area ratio (analyte peak area:IS peak area) against the drug concentration. Five calibrators for MET (10, 20, 40, 60, and 80 µg/mL) and six calibrators for PIO (10, 20, 40, 60, 80, and 100 µg/mL) were used. The method of least squares was used and, accordingly, the regression parameters were calculated. As a result, the correlation coefficients for MET and PIO were 0.9988 and 0.9985 respectively, indicating acceptable linearity [41,42]. The obtained values of the slope for MET and PIO were 0.0103 and 0.0388, respectively, indicating acceptable sensitivity as well. The standard deviations (SD) of the slopes for MET and PIO were 0.00028 and 0.00122, respectively. Low standard deviation values compared with the slope values of both drugs show low uncertainty in the regression analysis that may arise from the effect of indeterminate errors on the slopes [41,42]. The y-intercept values for MET and PIO calibrations were +0.0164 and −0.1603, respectively. In general, the ratio percent of the y-intercept with the variable data at nominal concentration are used to estimate the method variability. As a result, low levels of the calibration intercepts for both drugs suggest low level of variability in the proposed CZE method. Despite MET calibration recorded positive intercept, the small value suggests low level of the existence of interference with the response or low level of the saturation of responses at high concentrations. Similarly, the negative y-intercept of PIO at a small value enhances the claim of satisfactory sensitivity [41,42]. Furthermore, the standard deviation values of the y-intercepts for MET and PIO were 0.0140 and 0.0069, respectively. Low levels of standard deviations of both drugs indicate low level of uncertainty of calibrations [41,42]. Nevertheless, the uncertainty of MET is, to some extent, higher than that of PIO. Additionally, the standard error of the estimate values, or in another context the standard deviation of the regression, for MET and PIO were 0.01626 and 0.0876 for, respectively, indicating closer points to the calibration curve [41,42].

On the other side, the LODs (μg/mL) of MET and PIO were 0.091 and 0.277, respectively, whereas the LOQ (μg/mL) of MET and PIO were 0.104 and 0.315, respectively. It might be noticed that the LOD and LOQ of MET are slightly lower than those of PIO. Nevertheless, all levels are appropriate for the assay of both drugs in their pharmaceutical formulations as both drugs are active ingredients and present at high levels.

The intra-day precision was assessed by injecting five times in a day a standard mixture of MET and PIO at three different concentrations within the linear calibration curve, at 20, 40, and 60 μg/mL. The inter-day precision was also assessed by injection over a period of six days with standard mixture containing both drugs at 20, 40, and 60 μg/mL. The results are all compiled in Table 1. As shown, for intra-day precision, the relative standard deviation (RSD) values of migration times and corrected peak areas at all cases were less than 2.23% and 5.79%, respectively. For inter-day precision, the RSD values, at all cases, were less than 3.0% and 6.0% for migration time and corrected peak areas, respectively. In general, these results indicate good precision of the processed CZE method.

The accuracy of the CZE method was examined based on spiking in commercial tablet formulations. Several aliquots of the MET and PIO mixture at three different concentrations, namely, 20, 40, and 60 μg/mL, were added to a weighted, ground powder of the MET and PIO combination tablet. Table 2 shows that the recovery results for MET and PIO were 98.21–104.81 and 98.76–105.43%, respectively. These results indicate good accuracy of the method.

### 2.3. CZE Method Application

The proposed CZE method was applied to the determination of MET and PIO in the individual tablets and in a synthetic mixture as well as in their co-formulated tablets. The electrophoretic parameters including peak area, peak width, peak symmetry, and resolution are presented in Table 3. The recovery values, which was calculated based on the reported content, are also presented in Table 3.

The electropherograms of the applications of the CZE method to pharmaceutical formulations are illustrated in in Figure 3. As shown in Figure 3a, 75 µg/mL MET in Glucophage^®^ tables (500 mg MET) was successfully separated in the presence of 50 µg/mL IS. Figure 3b also shows successful separation of 30 µg/mL PIO in pharmaceutical formulation Actos^®^ tablets (30 mg PIO) in the presence of 50 µg/mL IS. These outputs demonstrate neither interference nor overlap of inactive ingredients of tablets formulations manufactures by different pharmaceutical industries with the single active ingredient of either MET or PIO. Furthermore, a synthetic mixture of 75 µg/mL MET, 50 µg/mL IS, and 30 µg/mL PIO exhibits successful separation as shown in Figure 3c. The electropherogram confirm the selectivity of the proposed CZE assay method for MET and PIO in the presence of inactive ingredients added at our laboratory. A sample solution of Actosmet^®^ included 75 µg/mL MET, 1.323 µg/mL PIO, and 50 µg/mL IS. Another sample solution of Actosmet^®^ included 1698 µg/mL MET, 30 µg/mL PIO, and 50 µg/mL IS. These two electropherograms confirm the selectivity of the CZE assay method for MET and PIO in real tablet formulation including different concentrations of active ingredients.

### 2.4. Comparison of CZE Method with HPLC Method

**Figure 4:**
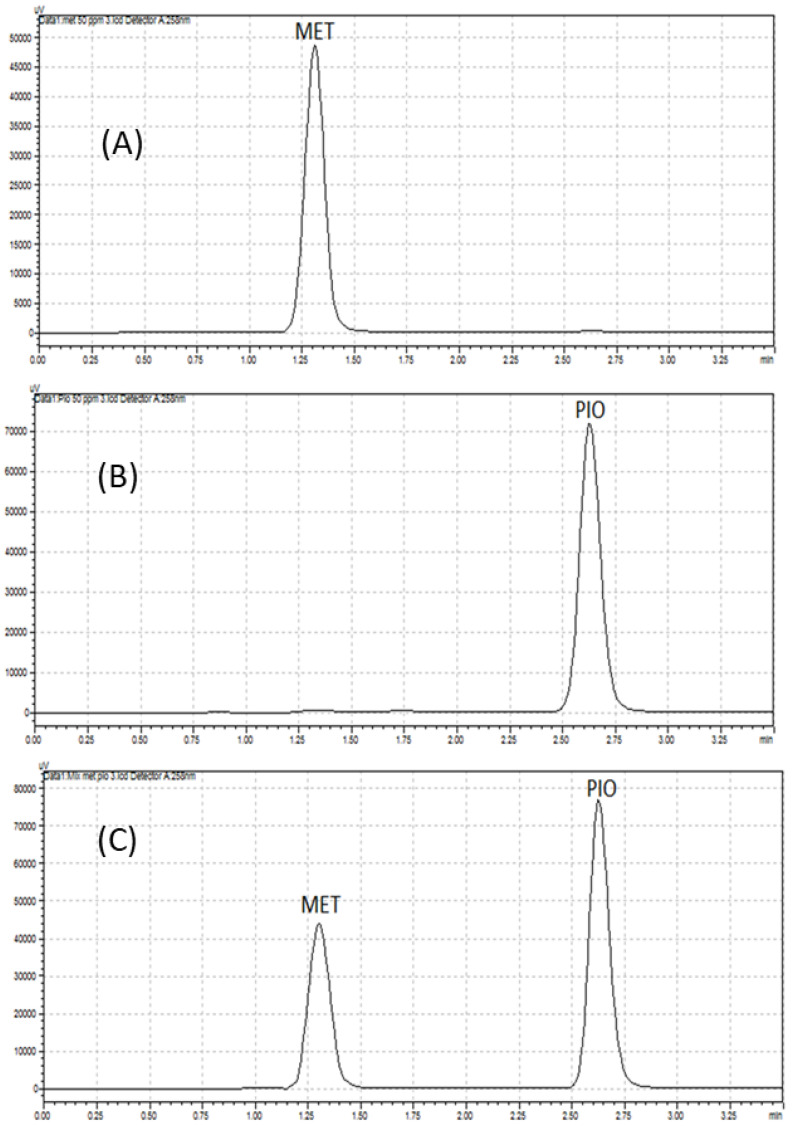
Typical chromatograms for application of high-performance liquid chromatography method for the analysis of (**A**) 50 µg/mL metformin (MET) in (glucophage^®^) (**B**) 80 µg/mL pioglitazone (PIO) in (actos^®^) (**C**) 50 µg/mL metformin (MET) and 80 µg/mL pioglitazone (PIO) in synthetic mixture.

A previous HPLC method [36] was applied for the assay of MET and PIO in their individual tablets and in synthetic mixtures, using the same reported conditions, with the exception of changing column length from 250 mm to 150 mm to obtain faster separation as well as the wavelength from 230 nm to 258 nm, which resulted in a shorter separation time. The chromatograms obtained for pharmaceutical formulations of MET and PIO are illustrated in Figure 4. The analytical features of both methods CZE and HPLC were compared and the results are summarized in Table 4. Both CZE and HPLC methods recorded successful separation with acceptable accuracy. The recovery values for the CZE method were 98.43–100.53%, while the recovery values for the HPLC method were 91.09–100.23%. Notably, the increased dispersion of HPLC is evidently observed by lower number of theoretical plates in HPLC than that of CZE. Lower level of the dispersion of the analytes obtained by the HPLC method than that obtained by the CZE method could also be observed in peak symmetry. While the peak symmetry of both analyte using HPLC was 1.00, the peak symmetry of MET and PIO using CZE were 1.00 and 1.30, respectively. Moreover, the RSD values expressing the intra-day and inter-day precision of CZE were higher than that of HPLC. Nevertheless, all RSD values did not exceed ≈5%, pointing out satisfactory precision of both methods. In general, weaker precision of CZE is likely associated with EOF fluctuations. and short optical path. In addition, the LODs and LOQs of the CZE method were lower than the HPLC method. As well, the recovery of MET is higher in CZE than HPLC, while the recovery of PIO is comparable with that in HPLC. In contrast, the run time of the HPLC method was shorter than the CZE method. Additionally, the HPLC system stabilization time (3 min) was shorter than that of the CZE (12 min).

It is noteworthy mentioning here that the CZE method consumed volumes of reagents and samples in microliters (0.0785), while the HPLC method consumed > 8 mL. Therefore, the CZE method is more economical and environmentally friendly than the HPLC method. Notably, several recent studies reported green analytical CZE methods for a wide range of applications including pharmaceutical, medicinal, food, and environmental analyses [43,44,45,46]. In general, the literature reported that CZE technique provided greener methods than HPLC technique [47].

## 3. Experimental

### 3.1. Chemicals and Reagents

Analytical-grade methanol (CH_3_OH) and acetonitrile (ACN) were purchased from Merck (Darmstadt, Germany). Deionized water was purified by a Milli-Q system (Millipore, New Bedford, Middlesex County, MA, USA) and was used throughout for the preparation of solutions. Orthophosphoric acid (85%), sodium phosphate dibasic heptahydrate, ammonia, hydrochloric acid, and sodium hydroxide were purchased from Sigma-Aldrich (Seelz, Germany). Standard materials of MET hydrochloride and PIO hydrochloride were supplied from Toronto Research Chemicals INC (North York, NY, Canada). Atenolol standard material was supplied from Sigma Aldrich, (Seelz, Germany). Glucophage^®^ tablets (500 mg MET) were prepared by Merck Serono. Actos^®^ (30 mg PIO) was prepared by Arab Pharmaceutical Manufacturing Co., Ltd., Amman, Jordan. Actosmet^®^ tablets (15 mg PIO /850 mg MET) were prepared by Jazeera Pharmaceutical Industries (JPI).

### 3.2. Instrumentation

Separations were conducted on a Beckman P/ACE MDQ capillary electrophoresis system. It is equipped with a UV radiation source, a selectable-wavelength UV/Vis photodiode detector (PDA) and a temperature-controlled sample storage module. The system is controlled by 32 Karat Software configured on a desktop controller. A fused-silica capillary column (Polymicro Technologies, Phoenix, AZ, USA) with a total length of 40 cm and effective length of 37 cm with an internal diameter (ID) of 52 μm was set up. The capillary was housed in a cartridge with a detector window of 100 × 800 µm (10 cm to the detector). Samples were hydrodynamically injected in the capillary at 0.5 psi for 10 s. The applied voltage was adjusted at 25 kV, while the temperature was set at 25 °C. The Thermo Scientific™ Orion™ 2-Star pH meter was used. The Shimadzu UV-1650, which is a calibrated double beam UV-visible spectrophotometer, was used as well. The Shimadzu prominence-*i* version named LC-2030 LT is one of models of the i-Series integrated HPLC and UHPLC systems family.

### 3.3. Preparation of Standard Solutions

A standard stock solution (1000 μg/mL) of MET was prepared in distilled deionized water, whereas a stock standard solution of PIO (1000 μg/mL) was prepared in methanol with the addition of few drops of HCl. Additionally, a standard stock solution of atenolol (1000 μg/mL), as an IS, was prepared in methanol. All solutions were sonicated and filtered through 0.45 μm PTFE Millipore filter. Mixed working standard solutions were prepared in the running buffer (75 mM phosphate buffer containing 30% ACN, pH 4) in an appropriate way at different concentrations ranged from 5 to 120 µg/mL for calibration purpose.

### 3.4. Preparation of Running Buffer

An amount of 200 mmol/L of sodium phosphate dibasic heptahydrate buffer was prepared as a stock solution, then diluted to different concentrations (20, 50, and 75 mmol/L) with ACN and distilled water. All solutions were sonicated and filtered through a 0.45 μm PTFE Millipore filter. The pH was adjusted with orthophosphoric acid 85%. The optimized running buffer solution was 75 mmol/L sodium phosphate buffer at pH 4.0.

### 3.5. Preparation of Pharmaceutical Samples

Ten tablets of each MET, PIO, and PIO-MET combination as previously described were weighed and powdered. Appropriate amounts were accurately weighed and transferred to 50-mL volumetric flasks. A MET tablet was dissolved in water, while PIO tablet and PIO-MET tablet combination were dissolved in methanol with the addition drops of HCl. A blank solution was synthesized for each individual drug. All solutions were sonicated and filtered through a 0.45 μm PTFE Millipore filter.

## 4. Conclusions

This study demonstrated the development of a CZE method for the separation and simultaneous determination of MET and PIO. All chemical and instrumental parameters presumed to be controlling the efficiency of the method were examined and optimized. Under the adopted optimum conditions, MET and PIO, besides the IS, were electrophoretic migrated and successfully separated in less than 4 min. After a rigorous comparison of CZE against HPLC, comparable features were obtained. Nevertheless, the CZE has the advantages of reduction of reagent consumption from milliliters to microliters and larger number of theoretical plates due to the use of capillary columns in electrophoresis technique. Therefore, the proposed CZE method is an appropriate alternative to be applied for routine analysis in pharmaceutical industry.

## Figures and Tables

**Table 1:** Relative standard deviation (RSD) values of repeatability and intermediate precision of the capillary zone electrophoretic assay method for MET and PIO.

Drug Content (μg/mL)	RSD% (Migration Time)		RSD% (Corrected Peak Area)	
	MET	PIO	MET	PIO
Intra-day precision				
20	0.74	0.49	5.79	5.03
40	1.42	2.23	5.45	0.81
60	1.36	1.98	3.36	4.31
Inter-day precision				
20	1.23	1.49	4.23	3.33
40	2.20	2.15	3.20	5.04
60	2.45	1.67	6.02	4.39

**Table 2:** Recoveries obtained from the determination of MET and PIO in commercial formulations that contain different levels of spiked standard.

Spiked Standard Mixture (μg/mL)	Recovery (%)	
	MET	PIO
20	104.8	105.4
40	100.5	99.6
60	98.2	98.8

**Table 3:** Application of the CZE assay method for MET and PIO in pharmaceutical formulations.

Sample	t_M_^a^		PH ^b^		PW ^c^		Res ^d^	PS ^e^		N ^f^		Rec ^g^	
	MET	PIO	MET	PIO	MET	PIO		MET	PIO	MET	PIO	MET	PIO
Glucophage^®^	2.31	-	8917	-	0.09	-	7.18	1.00	-	397.0	-	99.2	-
Actos^®^		3.35	-	6387	-	0.10	1.98	-	1.50	-	425.0	-	98.4
Synthetic mixture	2.30	3.44	11635	8065	0.10	0.09	1.64	1.00	1.00	394.9	449.7	98.8	98.4
Actosmet^®^	2.31	3.42	8917	8859	0.09	0.07	3.57	1.00	1.12	397.0	2417	100.5	99.6

a migration time (min), b peak area, c peak width, d resolution, e peak symmetry, f number of theoretical plates, g recovery.

**Table 4:** Comparison of the proposed capillary zone electrophoretic method with a high-performance liquid chromatographic (HPLC) method for the assay of metformin (MET) and pioglitazone (PIO).

Analytical Feature	CZE	HPLC
Separation capillary/column	Fused-silica capillary column (40 × 37 cm, 52 μm)	RP-18e column (150 × 4.6 mm, 5.0 μm) ^a^
Electrolyte/mobile phase composition	Sodium phosphate and 30% acetonitrile, pH 4	Sodium phosphate:acetonitrile (55:45), pH 5 ^a^
Consumed volumes of electrolyte/mobile phase (mL)	7.85×10^-4^	8.00
UV detection (nm)	PDA detector at 210	PDA detector at 258
Analysis time (min)	17.00	8.00
Waste production	In μLs	8.02 mL
System stabilization time (min)	12.00	3.00
Sample frequency (samples/h)	3	7
Retention/migration time (min) for MET and PIO	2.28 and 3.41	1.15 and 2.50
Resolution	10.37 between MET and IS; 1.64 between IS and PIO	2.07
Peak symmetry for MET and PIO	1.00 and 1.30	1.00 and 1.00
Theoretical plates for MET and PIO	2409.8 and 5388.9	221.8 and 533.1
Linear range (μg/mL) for MET and PIO	10–80 and 10–100	5–100 and 10–100
Correlation coefficient for MET and PIO	0.998 and 0.998	0.997 and 0.990
LOD (μg/mL) for MET and PIO	0.09 and 0.10	0.09 and 0.25
LOQ (μg/mL) for MET and PIO	0.27 and 0.31	0.29 and 0.76
Recovery (%) for MET and PIO	98.8–100.5 and 98.4–99.6	91.1–91.9 and 98.9–100.2
Intra-day precision (RSD%) for MET and PIO	3.35–5.78 and 0.81–5.02	0.21 and 0.25
Inter-day precision (RSD%) for MET and PIO	3.12–4.23 and 1.38–3.32	0.52 and 0.23

## Data Availability

The data presented in this study are available upon reasonable request from the corresponding author.

## Sample Availability

Not available.
